# Application of Information Link Control in Surgical Specimen Near-Miss Events in a South China Hospital: Nonrandomized Controlled Study

**DOI:** 10.2196/52722

**Published:** 2024-10-14

**Authors:** Tingting Chen, Xiaofen Tang, Min Xu, Yue Jiang, Fengyan Zheng

**Affiliations:** 1 Operating Room, The First Affiliated Hospital of Wenzhou Medical University, Nanbaixiang Street, Ouhai District, Wenzhou, Zhejiang Province, 325015, China, 86 13958929969

**Keywords:** near misses, technical barriers, process barriers, surgical specimens, information

## Abstract

**Background:**

Information control is a promising approach for managing surgical specimens. However, there is limited research evidence on surgical near misses. This is particularly true in the closed loop of information control for each link.

**Objective:**

A new model of surgical specimen process management is further constructed, and a safe operating room nursing practice environment is created by intercepting specimen near-miss events through information safety barriers.

**Methods:**

In a large hospital in China, 84,289 surgical specimens collected in the conventional information specimen management mode from January to December 2021 were selected as the control group, and 99,998 surgical specimens collected in the information safety barrier control surgical specimen management mode from January to December 2022 were selected as the improvement group. The incidence of near misses, the qualified rate of pathological specimen fixation, and the average time required for specimen fixation were compared under the 2 management modes. The causes of 2 groups of near misses were analyzed and the near misses of information safety barrier control surgical specimens were studied.

**Results:**

Under the information-based safety barrier control surgical specimen management model, the incidence of adverse events in surgical specimens was reduced, the reporting of near-miss events in surgical specimens was improved by 100%, the quality control quality management of surgical specimens was effectively improved, the pass rate of surgical pathology specimen fixation was improved, and the meantime for surgical specimen fixation was shortened, with differences considered statistically significant at *P*<.05.

**Conclusions:**

Our research has developed a new mode of managing the surgical specimen process. This mode can prevent errors in approaching specimens by implementing information security barriers, thereby enhancing the quality of specimen management, ensuring the safety of medical procedures, and improving the quality of hospital services.

## Introduction

### Overview

Pathological specimens are an important basis for judging the outcome of a patient’s disease [1]. Surgical specimen management involves many aspects, and errors in any part of the specimen management process may lead to serious consequences. The management of surgical pathological specimens directly affects the quality and safety of nursing management in the operating room. Therefore, how to reduce the occurrence of abnormal events has become the focus of nursing in the operating room.

A near miss is a commonly used term in clinical nursing, referring to a kind of nursing abnormal event that may damage the life, health, and safety of patients but has not yet developed to the end point [1,2]. It has similar causes and development paths with adverse events (the event caused inconvenience or harm to the patient), but the number is far greater than adverse events. At the same time, it has little harm to patients and hospitals and can give early warning before harm to patients, help the medical management system to carry out forward-looking and proactive risk assessment and prevention, which is regarded as a better learning resource and a risk management method advocated by the hospital’s fine management. Studies have shown that specimen approach failure is related to a variety of factors, such as human negligence, unreasonable workflow, and communication problems [3-7]. In recent years, information management of surgical specimens has gradually become the core issue that operating room managers continue to explore.

Surgical specimen informatization refers to the digital management process of specimen information obtained during surgery. It enhances data traceability, improves management efficiency, and ensures the accuracy and security of medical information. Specimen informatization involves digitally performing specimen collection, identification, transport, and reception [8]. Surgical specimen control refers to the effective management and control of specimen collection, processing, recording, and storage during surgery. It aims to ensure the quality of surgical specimens and data integrity. Process control should be implemented at critical nodes, with risk control points established throughout the entire process. Furthermore, problems occurring at all stages should be closely monitored and supervised [9].

### Study Purpose

In the quality and safety of nursing care in the operating room, it has always been a hot issue to explore the application of intelligent management to the surgical specimen inspection process and safety management [10]. The purpose of this study is to further explore the effectiveness evaluation and improvement methods of information safety barriers, and fill the gap in the research field of the quality and safety impact of surgical specimens. At the same time, a new model of surgical specimen process management is further constructed and a safe operating room nursing practice environment is created by intercepting specimen approach error events through information safety barriers.

## Methods

### Study Design and Participants

**Figure 1.**
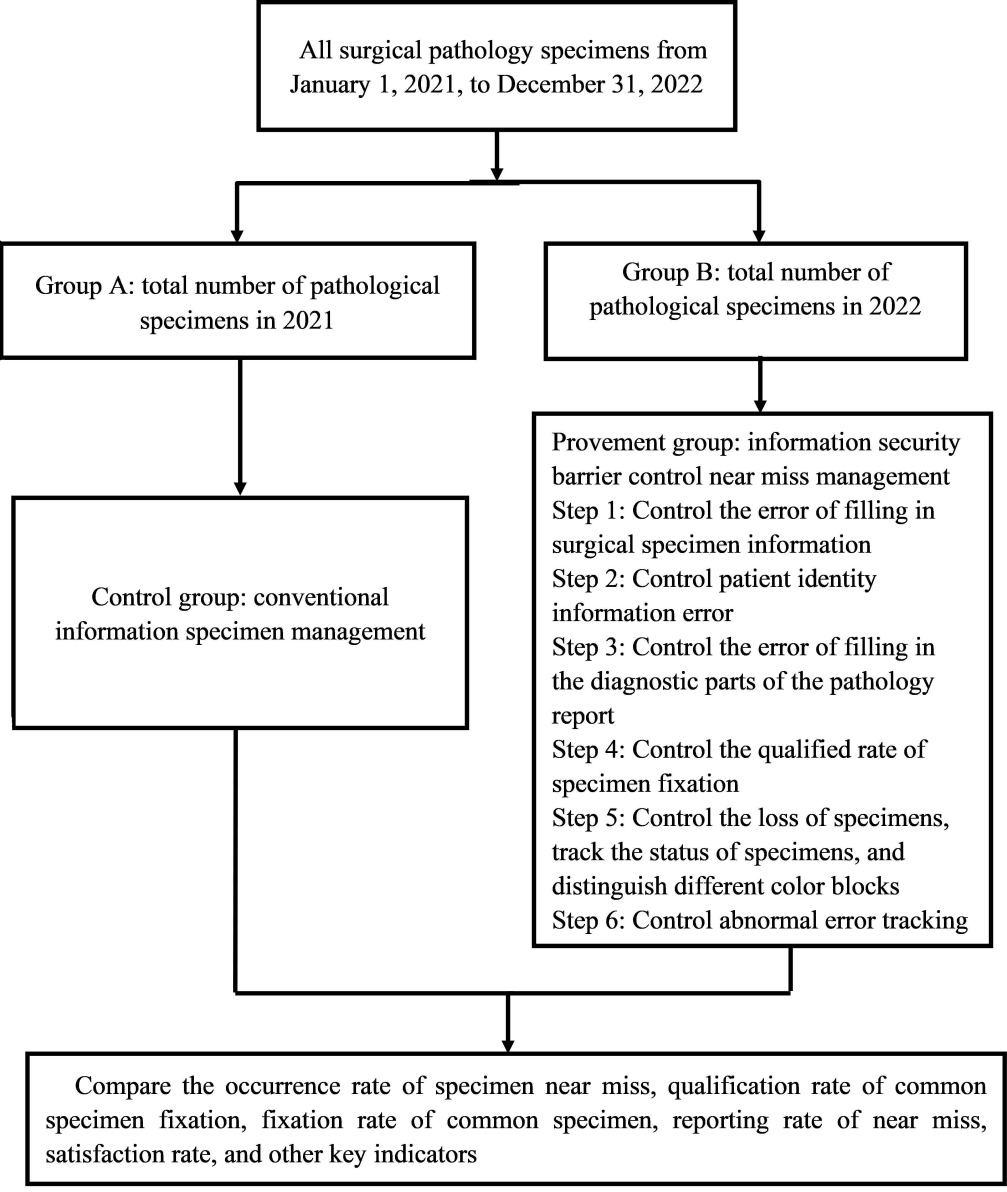
Flowchart of pathological sample test in operating room.

The objects of this study were different transportation modes of surgical specimens from 2021 to 2022. The acquisition and preliminary processing of surgical specimens were carried out by standardized trained surgeons and surgical nurses. The exclusion criteria for participants in this study were (1) there was no fixed time information for labeled specimens and (2) no specimen transport time was indicated. In this research, the information transportation process management model of surgical specimens in 2021 was classified as the control group (group A) and the improved information transportation process management model of surgical specimens in 2022 was classified as the experimental group (group B). The data related to these specimens were retrospectively analyzed to explore the advantages of the new mode over the traditional mode of information transportation process management of surgical specimens (Figure 1).

### Platform Run Time

The system uses a C/S (Client/Server) structure, using PowerBuilder (version 11.5; SAP) development tools and Oracle database (Oracle Corporation) development. It follows a 3-layer technical architecture mode, with each application module functioning independently to support multiuser concurrent operations. Users access the system through the computer client. The platform consists of 4 ports, each with specific components and functions illustrated in Table S1 in Multimedia Appendix 1.

**Figure 2.**
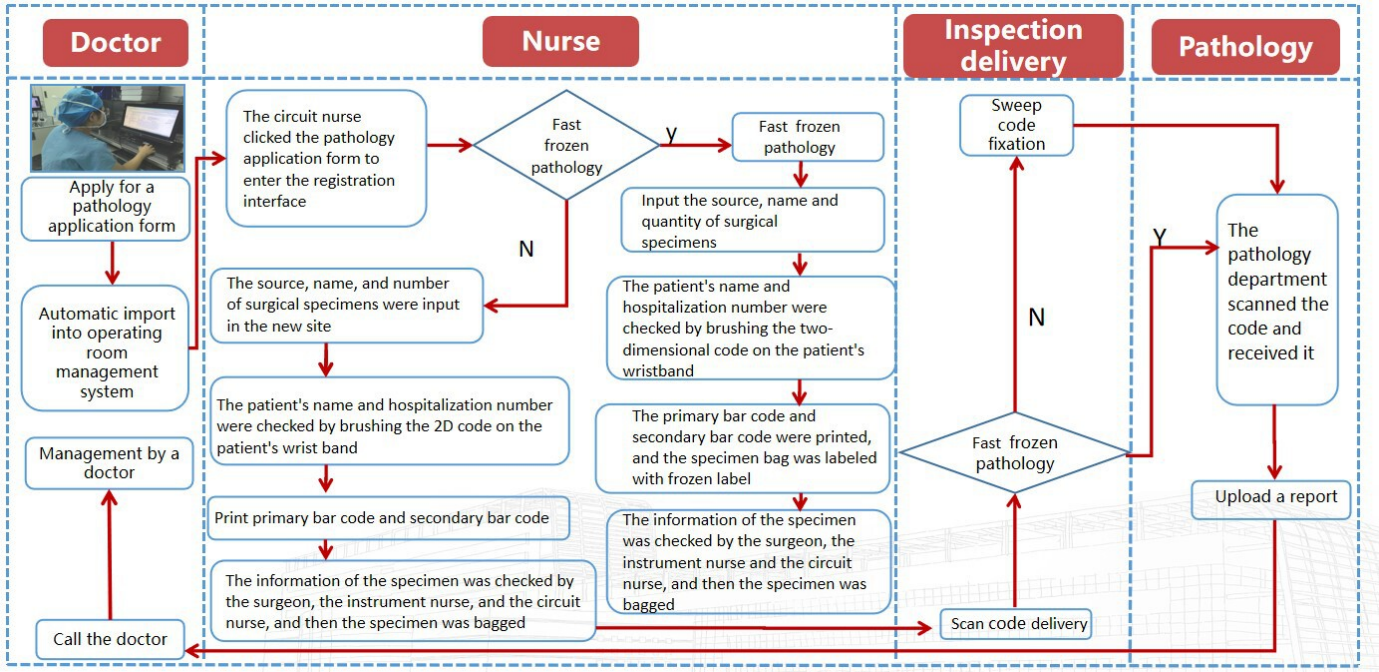
Flowchart of specimen inspection in the operating room.

Surgical specimen process management modes are first, label—handwashing nurse, itinerate nurse, and the attending surgeon tripartite the type and name of the surgical specimen sent for examination and print the primary and secondary barcodes of specimen information. Second, transport and fixation—dedicated surgical specimen delivery personnel. Finally, reception—the pathology department staff scanned the 2D barcode of the surgical specimen, received the surgical specimen, and scanned the code for traceability in all links. All links are scanned for traceability (Figure 2).

### Procedure for Each Port Specimen

#### Overview

The computer operation of the nurse in the operating room is as follows. First, the itinerant nurse selects the type of surgical specimen in the operation interface of the operating room management system nurse (Figure S1A in Multimedia Appendix 2). Then the itinerant nurse prints the first-level barcode (Figure S1B in Multimedia Appendix 2) and the second-level barcode (Figure S1C in Multimedia Appendix 2) of the specimen information and pastes the second-level barcode on the specimen while checking the type and name of the specimen confirmed by the handwashing nurse, the itinerant nurse, and the surgeon. Finally, the system automatically pops up a box to select the person who submitted for testing and pushes the corresponding specimen submission method and information into the operating room (Figure S1D in Multimedia Appendix 2).

The operation of sending and storing specimens on the hospital’s mobile app is as follows. First, the transport personnel used formalin for specimen fixation. Then the transport personnel scans the 2D barcode code on the surgical specimen on the hospital’s mobile app (Figure S1E in Multimedia Appendix 2) and confirms it (Figure S1F in Multimedia Appendix 2). Finally, the transport personnel sent the surgical specimen to the pathology department for confirmation.

#### Confirmation of Receiving Surgical Specimens in the Pathology Department

The personnel of the pathology department scan the 2D code of surgical specimens at the receiving window to confirm receipt and import into the pathology reporting system (Figure S1G in Multimedia Appendix 2).

#### Maintenance of Basic Items of the System and Approval of Pathological Application Form Modification

The operating room management system only allows the head nurse to approve the application form modification of incorrect surgical specimens. The manager of the pathology department can modify the application form approved by the head nurse and query the history record (Figure S1H in Multimedia Appendix 2).

#### Testing of Platform Functions

Before launching the platform, the research and development team repeatedly simulated tests to improve platform functions, simplify operation steps, beautify the appearance of the interface, increase the output traffic of the base station, and solve problems, such as slow network speed and running lag.

### Presurgical Specimen Analysis Stage

#### Link Control Step Diagram

The pictures below describe the control of each step in detail (Figure S2A in Multimedia Appendix 2).

#### Step 1

Control the error of filling in surgical specimen information. (1) Enter the operation specimen selection page and (2) select the specimen type (Figure S2B in Multimedia Appendix 2).

#### Step 2

Control patient identity information errors. (1) Label—surgical specimens, (2) the itinerant nurse enters the specimen’s name, (3) the system bounce window 1 prompts “Please scan the wristband 2D barcode code to confirm,” (4) the information is correct, and (5) the scan code is approved (Figure S2C in Multimedia Appendix 2).

#### Step 3

Control the error of surgical specimen information. (1) The system bounce window 2 prompts “The key information of the newly added surgical specimen site is inconsistent with the diagnostic site,” (2) the system automatic bounce box to please check with the surgical doctor again “Please enter the checker’s account password,” (3) double-check the information, (4) itinerant nurse input the surgeon’s account with the doctor’s consent, and (5) the surgeon can print the pathology bar code after confirming the information is corrects (Figure S2D in Multimedia Appendix 2).

#### Step 4

Control the qualified rate of specimen fixation. In the fixation process of ordinary pathological specimens, the system will automatically remind the unfixed surgical specimens after 20 minutes (Figure S2E in Multimedia Appendix 2).

#### Step 5

Control the error of the diagnosis site of the pathology report. Selecting the automatic import and retention of surgical specimens (Figure S2F in Multimedia Appendix 2).

#### Step 6

Control abnormal error tracking management of surgical specimens. The status of surgical specimens in each step is distinguished by different color blocks. The full-time specimen staff and the full-time receiving staff of the pathology department check whether each surgical specimen has reached the closed-loop state. If the surgical specimen is not received more than half an hour after isolation, the system will automatically warn that the specimen has not received the prompt. In case of surgical specimen execution error, the itinerant nurse should report the cause of the failure in applying for close proximity on the pathology application sheet interface, and the specimen should be correctly executed after the examination and verification by the leader nurse. Analyze and rectify each data fetching exception problem (Figure S2G in Multimedia Appendix 2).

### Primary Outcomes

The objective of this study is to compare the effectiveness of implementing information safety barriers in reducing the incidence of surgical specimen errors. These errors encompass inaccuracies in patient information registration, specimen identification and localization, as well as errors in the submission of specimens for examination. The error rate of surgical specimen’ submission is equal to the number of surgical specimen submission errors divided by the total number of surgical specimen submissions divided by 100%.

### Secondary Outcomes

This study aimed to compare the timeliness of fixing common pathological specimens before and after implementing the surgical specimen management mode with information security barrier control. The timeliness of fixation was assessed based on the passing rate and average fixation time of common pathological specimens. The fixed pass rate of common pathological specimens is equal to the number of fixed specimens completed within 30 minutes divided by the total number of fixed specimens multiplied by 100%. The average fixation time of common pathological specimens is equal to the total fixation time of a single common pathological specimen divided by the total fixation time of all common pathological specimens.

The reporting rate of surgical specimen near misses was compared between the 2 groups—the near miss events included wrong patient information registration, wrong specimen name and location, and wrong specimen type (such near miss events were corrected before the pathology report was issued by the pathology department, which did not cause serious consequences to patients, but there were great safety risks). The close error reporting rate of surgical specimens in the 2 groups was compared. The near error reporting rate is equal to the actual number of reported cases divided by the total number of reported close errors multiplied by 100%. The actual number of reported cases was the number of cases actually reported after the occurrence of abnormal events by revising the information on surgical specimens through written records.

The list of all surgeons and operating room nurses was obtained through the hospital information system, and 60 surgeons and 60 operating room nurses were randomly selected by using the random number table method to investigate the satisfaction of surgeons and operating room nurses on the surgical specimen disposal process and specimen management mode, and the satisfaction evaluation was collected at the end of specimen data collection in the control group and the improved group. Satisfaction is rated on a 5-point scale from very dissatisfied to very satisfied.

### Ethical Considerations

The study design and procedures conformed to the Declaration of Helsinki. This study was approved by the ethics review board of the First Affiliated Hospital of Wenzhou Medical University (KY2023-R098).

### Statistical Analysis

SPSS (version 26.0; IBM Corp) statistical software was used for statistical analysis. Use cases (percentage) and (mean, SD) were used to represent counting and measuring data, respectively. The *t* test was used for comparison among measurement data groups, the chi-square test was used for counting data, and the rank sum test was used for rank data.

## Results

### Primary Outcomes

From January 1, 2021, to December 31, 2021, the total number of pathological specimens is 84,289, of which 55,545 are ordinary, 1096 are paraffin accelerated, and 27,648 are frozen. From January 1, 2022, to December 31, 2022, the total number of pathological specimens was 99,998, of which 62,383 were ordinary, 5744 were paraffin accelerated, and 31,871 were frozen (Table 1). Before the implementation, there were 31 errors surgical specimen in submitting surgical specimens, including 17 errors in the name and location of surgical specimens, 6 errors in the type of specimens submitted, 2 errors in the information of registered patients, and 6 errors in the left and right sides of pathology reports. After the implementation, there were 4 errors in surgical specimens, including 4 errors in the name and location of surgical specimens (Table 2). The chi-square test showed that the error rate of surgical specimens’ submission was significantly lower than that before implementation and the difference was statistically significant (*χ*^2^_1_=25.9; *P*<.001).

**Table 1.** Summary table of surgical specimen types and quantities for 2021‐2022.

Year	Paraffin specimen, n	Frozen specimen, n	Paraffin acceleration, n	Total, n
2021	55,545	27,648	1096	84,289
2022	62,383	31,871	5744	99,998

**Table 2.** Information on the occurrence of misses from 2021‐2022.

Near miss events	Number (2021/2022), n/N	Key points of miss
Incorrect registration of patient information	2/0	Errors in electronic surgery notification checks
The specimen’s name was entered in the wrong place	17/4	Communication and checking failures between medical and nursing staff
Wrong type of surgical specimen sent for examination	6/0	Roving nurse operator error
Pathology report error	6/0	Pathologist makes error in entering surgical site in pathology report

### Secondary Outcomes

After the implementation of the new surgical specimen process management mode, the fixed pass rate of common pathological specimens increased to 99%, and the average fixation time was reduced to 10.221 (SD 12.552) minutes, while the differences were statistically significant (*P*<.001) with chi-square test and *t* test. At the same time, we found that only 13 of the 31 (41.9%) near-miss events in 2021 were reported, compared to all of the 4 (100%) near-miss events in 2022. We used Fisher precision probability test to measure the increase in the rate of near-miss reporting, which showed a statistically significant difference between 2021 and 2022 (*P*=.045; Table 3).

**Table 3.** Fixed qualification rate and average qualification time of common pathological specimens.

Indicators	2021	2022	Chi-square (*df*)/*t* test	*P* value
Fixed pass rate, n/N (%)	53,001/55,545 (95.5)	61,730/62,383 (98.9)	114,798.6 (1)^a^	<.001
Fixed meantime (minutes), mean (SD)	12.597 (13.032)	10.221 (12.552)	34.3 (232,657)^b^	<.001
The rate of near miss reporting, n	41.9	100	N/A^c^	.045

a Denotes the chi-square value.

b Denotes the *t* test value.

c N/A: not applicable.

Comparison of the satisfaction degree of doctors and nurses in relevant clinical departments with the procedure and management of surgical specimens before and after implementation. After the implementation, the satisfaction score of doctors in relevant clinical departments with the quality of pathological specimen management was 93.3% (56/60), higher than 58.3% (35/60) before the implementation, and the difference was statistically significant with rank sum test (*P*<.001; Table 4).

**Table 4.** Comparison of satisfaction with surgical specimen handling process and management between the 2 groups of medical staff.

Group		Great satisfaction, n	Satisfaction, n	Ordinary, n	Dissatisfaction, n	Be very dissatisfied, n	*z* score	*P* value
**Doctor satisfaction**							−5.133	<.001
	Control group (n=60)	15	20	22	2	1		
	Improvement group (n=60)	45	11	4	0	0		
**Nurse satisfaction**							−4.848	<.001
	Control group (n=60)	14	22	20	3	1		
	Improvement group (n=60)	46	10	4	0	0		

## Discussion

### Principal Findings and Contributions

The goal of this study is to develop a new surgical specimen operating standard and procedure that can effectively reduce the incidence of surgical specimen errors. Standardized procedures and standardized operations can simplify the work of nurses and medical teams and improve the quality of work [8]. In the traditional model mode, during the practice of the specimen identification stage, the surgeon’s verbal error, nursing interruption, input error, verification and communication failure, and other links would occur [11]. Key errors in surgical specimen management will directly lead to errors in surgical specimens, resulting in incomplete or inaccurate diagnoses of patients by clinicians. This can lead to inappropriate and inaccurate treatment and care, causing temporary or permanent physical and psychological harm to patients. Therefore, we have developed a new operating standard and procedure for surgical specimens, which can effectively reduce the occurrence of surgical specimen errors, greatly improve the working efficiency of the operating room, reduce the risk of surgical specimen route errors, and ultimately greatly improve the quality of medical care and guarantee the medical safety of patients.

There were 17 errors in identifying parts of surgical specimen names in our research in 2021, mainly on the left and right sides and the upper, middle, and lower parts. And the early warning information safety barrier in our new management model can intercept the wrong specimen names. When the specimen name is inconsistent with the diagnosis site during operation, the system will automatically pop-up to remind that the interception barrier is inconsistent with the diagnosis site of the patient (left and right side and other key controls such as upper, middle, and lower). If correct, the specimen name bar code will be printed. If it is inconsistent with the diagnosis site, the system will display the red large font box “specimen name is inconsistent with the diagnosis, please check the specimen name again is correct” to play a role in checking again. In particular, errors on the right and left sides of key points can lead to a series of pathological diagnosis errors leading to very serious adverse events. The results showed that in 2022, the error rate of specimen name identification was significantly reduced to 4 cases, among which 4 cases of specimen name error were mainly caused by the failure of bilateral surgical site verification for the same incision. Meanwhile, the error rate of surgical specimens was significantly lower than that before the operation (*χ*^2^_1_=25.9, *P*<.001).

### Theoretical and Practical Significances

In the quality and safety of nursing care in the operating room, it has always been a hot issue to explore the application of intelligent management to the surgical specimen inspection process and safety management [10]. The process of specimen inspection was improved to achieve information-based closed-loop management, and barcode technology was used to track and record the whole process of specimen inspection to form closed-loop management, so as to improve the accuracy and traceability of intraoperative pathological specimen information [9]. The traditional specimen inspection is done by scanning the 2D barcode code, but there is no information reminder function. In this paper, the link of specimen fixation is proposed. If the surgical specimen is not fixed 20 minutes after registration, the computer pop-up window will remind “Abnormal specimen submission,” display the name of the corresponding operating room, itinerant nurses and patient information, and urge the itinerant nurses to submit pathological specimens for examination in time to prevent late and missed inspection of surgical specimens. Barcode technology is used to track and record the whole process of surgical specimen submission, forming closed-loop management and improving the accuracy and traceability of intraoperative pathological specimen information [12], accurately implement closed-loop management of specimens, transport personnel pays attention to the status of surgical specimens in real time, whether it has closed loop, and promptly urge itinerant and hand-washing nurses to implement the specimen inspection work in strict accordance with the surgical specimen inspection system. If the time from marking to receiving of surgical specimen exceeds half an hour, the system will show a red warning module that the specimen is not alerted to the pathology department, which can effectively reduce the incidence of surgical specimen delivery near misses. A shorter time for surgical specimen examination can reduce the difficulty of pathological diagnosis [13]. The qualified rate of fixation of common pathological specimens increased to 99%, and the differences were statistically significant (*P*<.001). Controlling the closed-loop management of the information of each link of surgical specimens, the number of specimens sent for surgery at each stage, and the status of specimens at each link are distinguished by different color blocks. The full-time specimen-sending personnel and the full-time receiving personnel of the pathology department checks whether each surgical specimen has reached the closed-loop state. It achieves accurate control of each link in the process of submitting surgical specimens, refines the standardized process of submitting surgical specimens, and ensures the controllability of each link, thus ensuring the correctness and simplification of the entire process of submitting surgical specimens, improving the qualification rate of handling surgical specimens, and reducing the risk of approaching errors [14].

With the high-quality development of hospitals, the informatization of operating rooms has been fully popularized and entered the era of a new paperless model [15]. It is particularly important to track and manage the surgical specimens and analyze the abnormal process. The traditional mode refers to the manual way to collect information manually, and the information filled in manually may have problems such as missing filling, wrong filling, mistransmission, and so forth. This paper uses network technology to realize information sharing and captures data through nursing managers to modify surgical specimens and near misses. In case of surgical specimen execution error, the traveling nurse should report the cause of the incident near the error on the interface of the pathology application form, and the specimen will be correctly executed after approval and verification by the head nurse. After using the new management mode, the near-miss reporting rate increased to 100% (*P*=.045). Meanwhile, this is also one of the ways to collect surgical specimen errors. Through information collection, we can find the causes of specimen errors and analyze and improve the key links of information. In the event of a surgical specimen near miss, the nurse on duty applied for specimen review, entered the reasons for the error, and made an application. After the review by the regulator, the correct name of the specimen can be obtained, the analysis and rectification of each data capture abnormal problem can be carried out, and scientifically use the quality tracking management tool of specimen approaching error anomaly to analyze the root cause and learn from it. It is helpful to improve the correct execution rate of surgical specimens and enhance the awareness of specimen management [2].

IT has been used to effectively improve the process of surgical specimen inspection [16], which has a sensitive and accurate perception function and effective verification, supervision, and control function, and improves work efficiency and the overall quality of specimen inspection. The satisfaction of medical staff had significantly increased to 93.3% (*P*<.001), and the efficiency of surgical specimen inspection was improved. The research on the prevention of surgical specimens near misses through effective information control link technology and the establishment of intelligent closed-loop management mode of pathological specimens in intelligent operating rooms is still rare, and there are no relevant reports at home and abroad. Through statistical analysis of medical satisfaction, this paper shows that the work of nurses and medical teams can be simplified and the quality of work can be improved [7,8]. The purpose of this study is to construct a new model of surgical specimen process management through the information security barrier to prevent the specimen from approaching the wrong event to create a safe operating room nursing practice environment.

### Limitations

First, different personnel receive different education levels and ages, so different personnel have different ability to operate the system, so there will be deviations. Furthermore, different types of surgery may require different methods of recording and managing specimen information. There is also a Hawthorne effect in this study, which affects the results. In addition, inadequate information management systems can lead to data underreporting or inaccurate reporting, which can have serious implications for patient diagnosis and treatment. Finally, the direct relationship between the management model and patient outcomes was not discussed in our study and we will conduct this part in future studies. In conclusion, all of these issues may lead to limitations in the study.

### Conclusions

We have developed a novel mode of managing the surgical specimen process. This new model effectively controls and manages the entire process, including the preanalysis stage, analysis stage, and fault analysis stage, of the information security barrier. It significantly reduces the risk of near misses associated with surgical specimens. Moreover, the new management model serves as a valuable reference for clinical decision makers and enables multiple hospitals to enhance operating room efficiency, reduce the occurrence of near misses in surgical specimens, and ultimately improve the quality of medical care while ensuring patient safety.

## Supplementary material

10.2196/52722 Multimedia Appendix 1 Module.

10.2196/52722 Multimedia Appendix 2 Procedure for each port specimen.
