# Supplementary material for: Application of Information Link Control in Surgical Specimen Near-Miss Events in a South China Hospital: Nonrandomized Controlled Study
Source: JMIR Med Inform. 2024 Oct 14;12:e52722. doi: 10.2196/52722 (PMC11492967; doi:10.2196/52722)
Supplement: Multimedia Appendix 1 [file medinform-v12-e52722-s001.docx]

| Table S1. Module. | | |  |
| --- | --- | --- | --- |
| Port | Module | Feature | |
| Nurse | Pathology application form registration | Mark specimen information, print specimen two-dimensional barcode, reject processing, abandon application, etc | |
|  | Statistical query of pathological specimens | The specimen information can be queried according to the marking time, inspection type, application source, specimen status, and inspection type | |
|  | Application for modification of pathology application form | The pathology department has received the specimen, the nurse can apply to modify the type of examination, specimen site, or abandon the application | |
|  | Examination and approval of pathological application form | The head nurse has the authority to approve the application form modification application | |
| Transport personnel | Confirmation of delivery/delivery of pathological specimens | The two-dimensional code of the specimen was scanned for delivery confirmation, and the specimen was sent to the pathology department for delivery confirmation | |
| Pathology department | Pathological specimens were received and confirmed | The receiving window of the pathology department scanned the two-dimensional code of the specimen for acceptance confirmation and import into the pathology reporting system. The problematic application forms were rejected | |
|  | Pathological application form modified | Modify approved application forms and query historical records | |
| System administrator | System basic project maintenance | Maintain basic projects related to the system | |
|  | Pathology request item maintenance | Maintain specimen sites and charges according to the source | |
