# Supplementary material for: Application of Information Link Control in Surgical Specimen Near-Miss Events in a South China Hospital: Nonrandomized Controlled Study
Source: JMIR Med Inform. 2024 Oct 14;12:e52722. doi: 10.2196/52722 (PMC11492967; doi:10.2196/52722)
Supplement: Multimedia Appendix 2 [file medinform-v12-e52722-s002.docx]

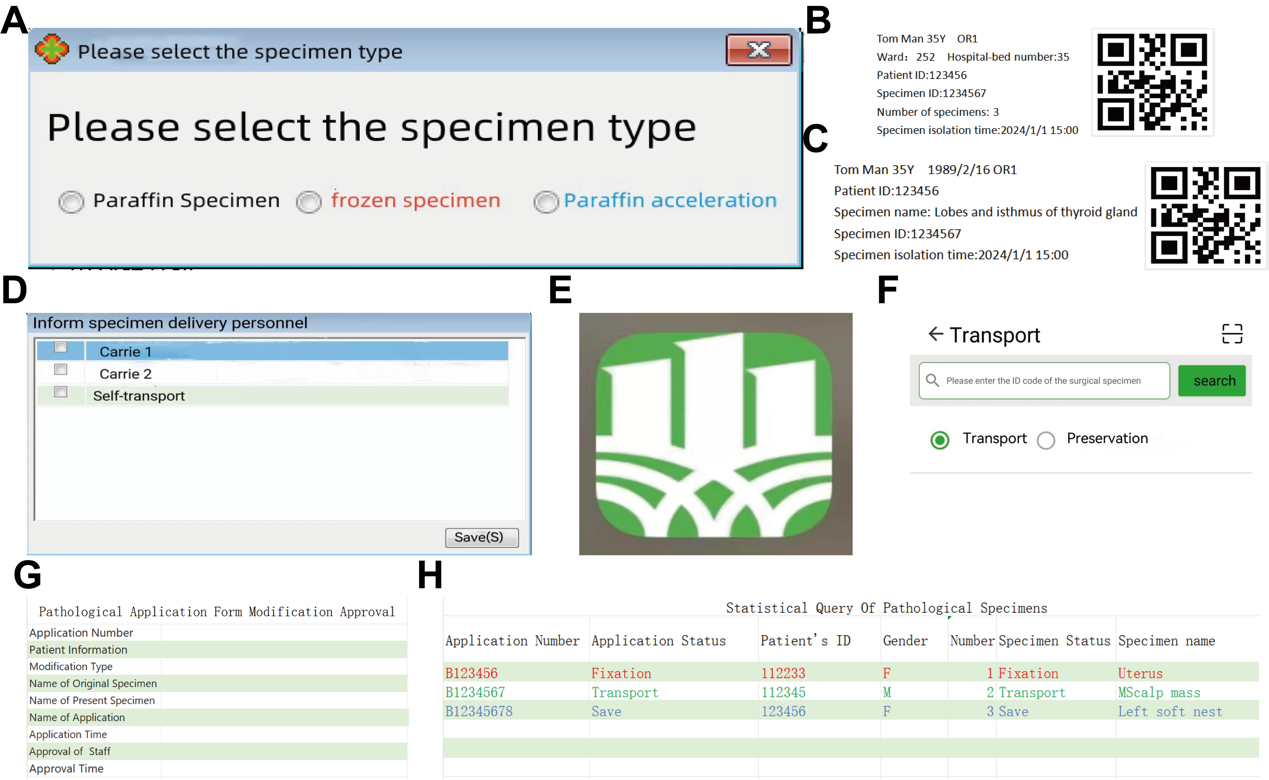


**Figure S1. A.** Select surgical specimen type. **B**. Main bar code for specimen inspection. **C**. Barcode for sample delivery. **D**. Specimen delivery and display. **E**. Mobile APP. **F**. Mobile phone scanning specimen transport and specimen preservation. **G**. Pathology reporting system. **H**. Approval of pathological application form modification.


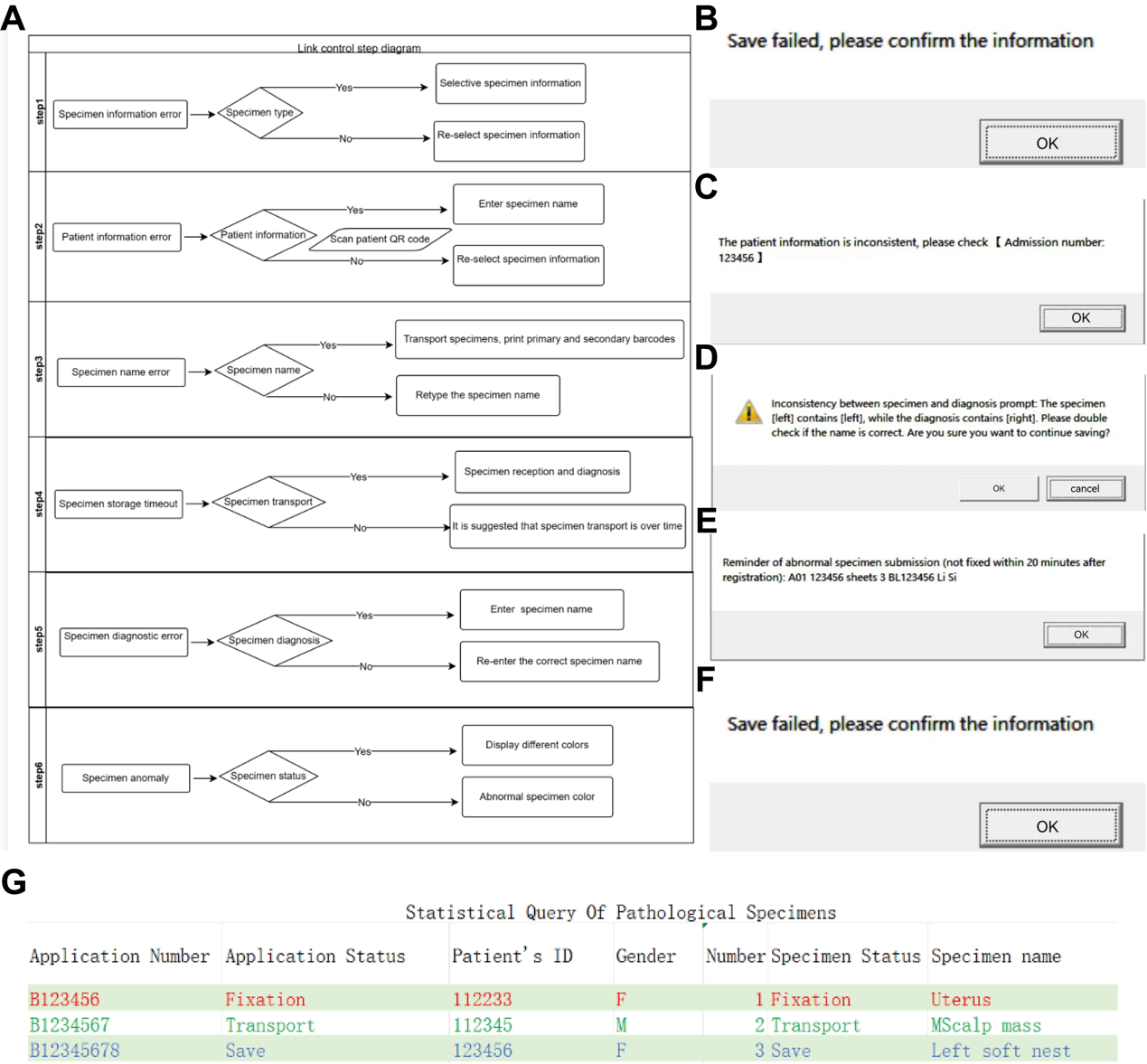


**Figure S2. A.** Link control step diagram. **B**. Control the error of filling in surgical specimen information. **C**. Control patient identity information errors. **D**. Control the error of surgical specimen information **E**. Control the qualified rate of specimen fixation. **F**. Control the error of Position error. **G**. Control abnormal error tracking management of surgical specimens.
